# Variation and transmission of the human gut microbiota across multiple familial generations

**DOI:** 10.1038/s41564-021-01021-8

**Published:** 2021-12-30

**Authors:** Mireia Valles-Colomer, Rodrigo Bacigalupe, Sara Vieira-Silva, Shinya Suzuki, Youssef Darzi, Raul Y. Tito, Takuji Yamada, Nicola Segata, Jeroen Raes, Gwen Falony

**Affiliations:** 1 Laboratory of Molecular Bacteriology, Department of Microbiology and Immunology, Rega Institute, Katholieke Universiteit Leuven, Leuven, Belgium; 2 Center for Microbiology, Vlaams Instituut voor Biotechnologie, Leuven, Belgium; 3 School of Life Science and Technology, Tokyo Institute of Technology, Tokyo, Japan; 4 European Institute of Oncology Istituto di Ricovero e Cura a Carattere Scientifico, Milan, Italy; 5 Present Address: Department for Integrative Biology, University of Trento, Trento, Italy

**Keywords:** Metagenomics, Microbiome

## Abstract

Although the composition and functional potential of the human gut microbiota evolve over the lifespan, kinship has been identified as a key covariate of microbial community diversification. However, to date, sharing of microbiota features within families has mostly been assessed between parents and their direct offspring. Here we investigate the potential transmission and persistence of familial microbiome patterns and microbial genotypes in a family cohort (*n* = 102) spanning 3 to 5 generations over the same female bloodline. We observe microbiome community composition associated with kinship, with seven low abundant genera displaying familial distribution patterns. While kinship and current cohabitation emerge as closely entangled variables, our explorative analyses of microbial genotype distribution and transmission estimates point at the latter as a key covariate of strain dissemination. Highest potential transmission rates are estimated between sisters and mother–daughter pairs, decreasing with increasing daughter’s age and being higher among cohabiting pairs than those living apart. Although rare, we detect potential transmission events spanning three and four generations, primarily involving species of the genera *Alistipes* and *Bacteroides*. Overall, while our analyses confirm the existence of family-bound microbiome community profiles, transmission or co-acquisition of bacterial strains appears to be strongly linked to cohabitation.

## Abstract (web summary)

Quantitative metagenomic analyses of gut microbiomes reveals kinship, together with current cohabitation, as drivers of microbial community transmission and persistence between family members over three to five generations.

## Main

The characterization of the acquisition and maturation of the human gut microbiota over the lifespan is of key importance for future clinical translation of microbiome research. Assessing transmissibility of bacterial strains and determining whether they are passed on at birth or acquired only later in life will support the development of guidelines to facilitate or hamper transmission depending on their beneficial or risk profile, respectively^1^. Based on such a timeline and depending on whether the acquisition of a specific strain should be considered a health benefit or rather a risk factor with respect to disease development, guidelines to facilitate/hamper transmission can be formulated^1^. Given reports of maternal inheritance of microbial strains^2–5^, strain sharing among individuals sharing households^6^ and transmission events spanning multiple generations in animal models^7–9^, similar considerations might apply when assessing the familial burden of conditions with a potential microbiota contribution, ranging from obesity^10^ to inflammatory bowel diseases^11^.

## Results and Discussion

### Microbiome variation in a multigenerational family cohort is associated with age

**Fig. 1:**
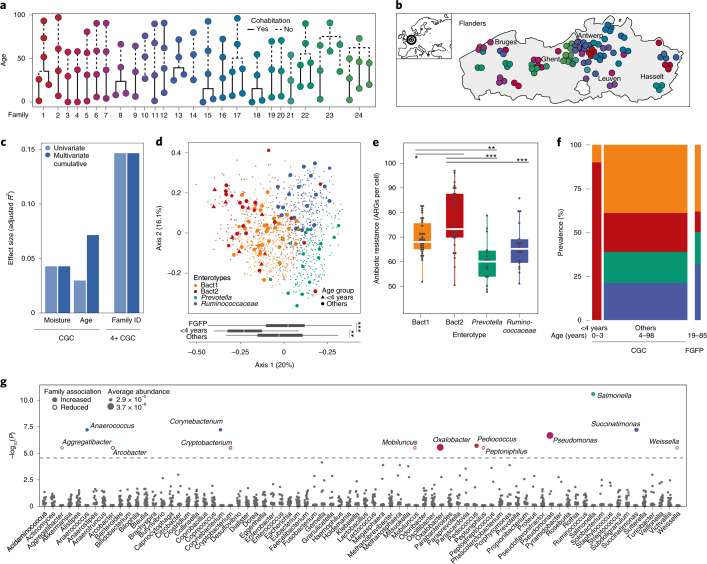
Familial structures and microbiome profiles. **a**, Family structures in the CGC cohort (*n* = 102). The colours represent family IDs and the line type indicates current cohabitation status. **b**, Geographical distribution of the participants across Flanders (Belgium). Family IDs are coloured as in Fig. 1a. **c**, Cumulative effect sizes of significant covariates on microbiome community variation (dark blue, right bars; stepwise dbRDA on Bray–Curtis dissimilarity) compared to individual effect sizes assuming covariate independence (light blue, left bars; dbRDA on Bray–Curtis dissimilarity) in the CGC (*n* = 101) and the ≥4-year-old CGC (4+ CGC, *n* = 91) cohorts (Supplementary Table 2). Age, SBMI, delivery mode, family ID, cohabitation status, medication use, antibiotic use, moisture content (%) and faecal calprotectin (μg g^−1^) were tested as potential microbiome covariates. **d**, PCoA of interindividual differences (Bray–Curtis) in relative microbiome profiles of the CGC cohort (*n* = 101 samples, larger dots) on a background dataset from a cross section of the Flemish population (*n* = 1,106 samples, small dots). The distribution of samples along the first axis of the PCoA (bottom box plots) separated young children (<4 years old, *n* = 10) from other individuals (*n* = 91) and FGFP samples (*n* = 1,106); Kruskal–Wallis with post-hoc Dunn test, **P_adj_< 0.01, ***P_adj_ < 0.001. The body of the box plot represents the first and third quartiles of the distribution and the median line. The whiskers extend from the quartiles to the last data point within 1.5× the interquartile range (IQR), with outliers beyond. Bact1: *Bacteroides* 1; Bact2: *Bacteroides* 2. **e**, Increased ARG load (*n* ARG/microbial load in Bact2 enterotyped samples; *n* = 101, Kruskal–Wallis test, chi-squared = 26.7, *P* = 6.8 × 10^−6^; post-hoc Dunn test, *P_adj_ < 0.05, **P_adj_ < 0.01, ***P_adj_ < 0.001; Supplementary Table 3. The body of the box plot represents the first and third quartiles of the distribution and the median line. The whiskers extend from the quartiles to the last data point within 1.5× the IQR, with outliers beyond. Colour coding as in Fig. 1d. **f**, Increased prevalence of the Bact2 enterotype in young children (*n* = 10) compared to other individuals (*n* = 91) and FGFP samples (*n* = 1,106); pairwise chi-squared test, *P*_adj_ < 0.1; Supplementary Table 4. Colour coding as in Fig. 1d. **g**, Seven bacterial genera displayed significantly higher abundances among members of specific families compared to the rest of the 4+ CGC dataset (*n* = 91, two-sided Wilcoxon rank-sum test, −log_10_(*P*) > 4.56; Supplementary Table 5). The colours correspond to family IDs (as in Fig. 1a), while the circle sizes are proportional to the average abundance of the genus. The closed circles indicate genera with significantly increased abundances in a specific family; the open circles indicate those with decreased abundances in specific families.

To explore the persistence of transmittable microbial features across generations in the human host, we assembled a unique dataset of stool samples from women belonging to 24 multigenerational families living in the region of Flanders (Belgium) with accompanying metadata covering anthropometrics, delivery mode, cohabitation status, levels of systemic and local inflammation markers and use of medication (Fig. 1a,b and Supplementary Table 1). One hundred and two healthy individuals (aged 0–98, median = 37.5, born between 1917 and 2016) were sampled between November 2015 and November 2016. The standardized body mass indices (SBMI) of participants (an age- and sex-corrected version of the body mass index valid also in children^12,13^) varied between 7 and 56 (median = 37) with most individuals falling within the normal range (*n* = 60 out of 87; normal range 30–39). Ninety-nine (*n* = 99 out of 102) were born by vaginal delivery. Family structures ranged from 3 up to 5 generations (median = 4), presenting different degrees of multigenerational cohousing and geographical dispersion.

Exploring host or environmental factors significantly contributing to interindividual microbiome variation in our cross-generational cohort (CGC), we combined shotgun metagenomic sequencing data with flow cytometry measurements of faecal microbial load^14^ to construct quantitative microbial abundance profiles. Within the limitations of the CGC cohort, stool moisture (*n* = 101, stepwise distance-based redundancy analysis (dbRDA) at the genus-level Bray–Curtis dissimilarity, *R*^2^ = 4.3%, *P*_adj_ = 2 × 10^−4^) and age (*R*^2^ = 2.9%, *P*_adj_ = 2 × 10^−4^) were identified as the only metadata variables with non-redundant explanatory power over quantitative microbiome variation (Fig. 1c and Supplementary Table 2). These findings align with previous reports on proportional microbiome variation in population cohorts^15^, with stool moisture, a proxy of colonic transit time^16^, reflecting ecosystem development induced by nutrient depletion on passage through the gastrointestinal tract^17^. Additionally, we confirmed the negative associations between faecal water content and microbial load (*n* = 101, Spearman’s test, *ρ* = −0.25, *P* = 1.2 × 10^−2^) as well as genus-level microbiome richness (*n* = 101, *ρ* = −0.29, *P* = 3.7 × 10^−3^)^18^. Although 21 participants reported to have taken antibiotics during the 12 months before sampling, we did not observe a significant impact of (history of) antibiotic therapy on microbiome composition in the present CGC cohort (Supplementary Table 2). Following up on reports of altered microbial ecosystem configurations in early childhood^19,20^, we assessed a potential association between age bins (young children <4 years old, *n* = 10 versus others (≥4 years old CGC, 4+ CGC), *n* = 91) and quantitative genus-level microbiota composition. In a multivariate model, age bins were shown to have the largest effect size (*n* = 101, genus-level stepwise dbRDA, *R*^2^ = 8.0%, *P*_adj_ = 1 × 10^−4^; Supplementary Table 2), with moisture contributing an additional 1.7% to microbiome variance (*P*_adj_ = 1.1 × 10^−2^). Hence, we confirmed that young children harbour a markedly different microbiota when compared to individuals with a fully matured colon ecosystem^19,20^.

### The *Bacteroides*2 enterotype is highly prevalent among young children

**Extended Data Fig. 1:**
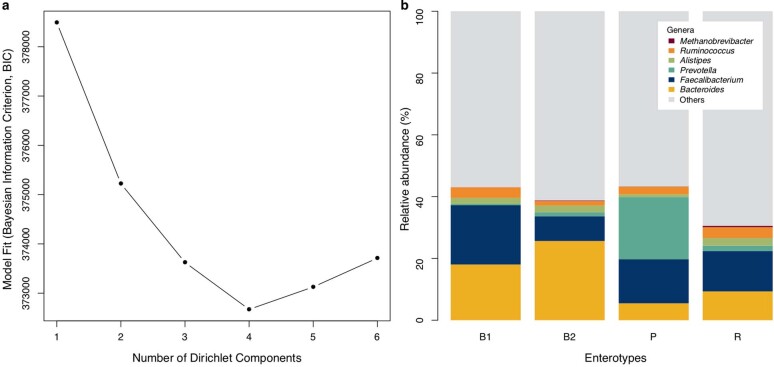
Enterotype stratification by DMM community typing. (**a**) Identification of the optimal number of clusters (Dirichlet components) in the CGC dataset (N = 101) complemented with 1106 samples from the FGFP^26^ cohort based on the Bayesian Information Criterion (BIC). (**b**) Barplot representation of the average relative abundance of a few representative genera split into the four enterotypes identified by DMM community typing on the combined CGC and FGFP sets (N = 1207).

**Extended Data Fig. 2:**
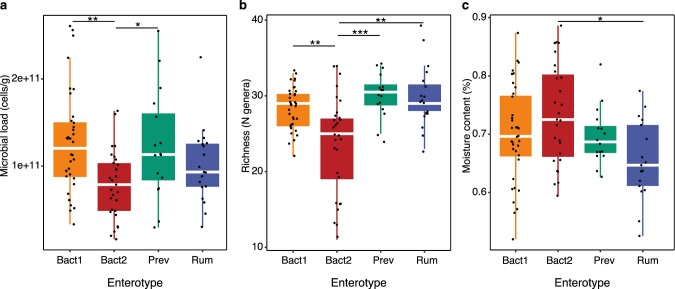
Enterotype associations with microbial load, richness, and moisture content. (**a**) Reduced microbial load (microbial cells per gram of stool) in Bact2 enterotyped samples; N = 101, KW test Chi^2^ = 13.9, P = 3.0e-03; phD tests, adjP<0.001(***), <0.01(**), <0.05(*); Supplementary Table 3. (**b**) Reduced richness (number of genera) in Bact2 enterotyped samples; N = 101, KW test Chi^2^ = 20.0, P = 1.6e-04; phD tests, adjP<0.001(***), <0.01(**), <0.05(*); Supplementary Table 3. (c) Increased stool moisture content (%) in Bact2 enterotyped samples; N = 101, KW test Chi^2^ = 8.8, P = 0.03; phD tests, adjP<0.001(***), <0.01(**), <0.05(*); Supplementary Table 3. The body of all box plots represent the first and third quartiles of the distribution and the median line. The whiskers extend from the quartiles to the last data point within 1.5× the interquartile range, with outliers beyond.

Recently, we identified a faecal microbiota community type with high prevalence in cohorts of individuals with obesity^21^, inflammatory bowel disease^14,22^ and primary sclerosing cholangitis^22^, as well as among individuals with certain subtypes of multiple sclerosis^23^ and depression^24^. Common features of this potentially dysbiotic *Bacteroides*2 (Bact2) enterotype include low compositional richness, low faecal cell counts and high and low proportional abundances of the *Bacteroides* and *Faecalibacterium* genera, respectively. In general, Bact2-enterotyped individuals present looser stools and higher (both intestinal and systemic) inflammation markers^22^. To distinguish community states within the present CGC, we performed Dirichlet multinomial mixture (DMM) modelling^25^ against the background of microbiome variation as observed in the Flemish Gut Flora Project (FGFP) dataset (*n* = 1,106 population cohort)^26^. To this end and to preclude community clustering driven by methodological differences, the CGC dataset was additionally profiled using 16S ribosomal RNA gene amplicon sequencing following FGFP procedures^26^. The resulting amplicon profiles were only used for the purpose of enterotyping. Applying probabilistic models to group samples potentially originating from the same community, DMM-based stratification reproducibly identifies microbiome configurations across datasets without making any claims regarding the putative discrete nature of the strata detected. Microbiomes were observed to stratify over four previously described enterotypes^14^, labelled as *Bacteroides*1 (Bact1), Bact2, *Prevotella* and *Ruminococcaceae* (Fig. 1d and Extended Data Fig. 1). Bact2 samples diverged from their non-Bact2 counterparts, displaying lower microbial load (*n* = 101, Kruskal–Wallis test, chi-squared = 13.9, *P* = 3.0 × 10^−3^; post-hoc Dunn test, *P*_adj_ < 0.05 for Bact2 versus Bact1/*Prevotella*), lower genus-level richness (*n* = 101, Kruskal–Wallis test, chi-squared = 20.0, *P* = 1.6 × 10^−4^; post-hoc Dunn test, *P*_adj_ < 0.05 for Bact2 versus Bact1//*Prevotella*/*Ruminococcaceae*) and higher stool moisture content (*n* = 101, Kruskal–Wallis test, chi-squared = 8.8, *P* = 0.03; post-hoc Dunn test, *P*_adj_ < 0.05 for Bact2 versus Ruminococcaceae; Extended Data Fig. 2 and Supplementary Table 1). With only a single participant scoring above the serum C-reactive protein (CRP) clinical threshold (>15 mg l^−1^) and 11 above the faecal calprotectin one (>200 μg g^−1^; Supplementary Table 1)^27^, we did not observe higher prevalence of systemic nor intestinal inflammation among participants hosting Bact2 (*n* = 77, chi-squared = 1.8, *P* > 0.05; *n* = 99, chi-squared test = 7.1, *P* > 0.05) in contrast with previous reports regarding associations in specific patient groups^22^. Although enterotype stratification was not significantly associated with participants’ history of antimicrobial drug intake (*n* = 95, chi-squared = 5.0, *P* > 0.05), low microbial load Bact2 samples were proportionally enriched in antimicrobial resistance genes (ARGs; *n* = 101, Kruskal–Wallis test, chi-squared = 26.7, *P* = 6.8 × 10^−6^; post-hoc Dunn test, *P*_adj_ < 0.05 for Bact2 versus Bact1/*Prevotella*/*Ruminococcaceae*; Fig. 1e and Supplementary Table 3). Enterotype distribution in the CGC cohort differed significantly from the proportions observed in the FGFP population cohort (*n* = 101 versus *n* = 1,106, chi-squared test = 25.9, *P* = 9.98 × 10^−6^), with the present dataset being characterized by a higher prevalence of Bact2 samples (29% versus 12%; pairwise chi-squared test = 22.3, *P*_adj_ = 9.1 × 10^−6^; Supplementary Table 4). More specifically, young children (<4 years old) displayed a markedly higher prevalence of Bact2 configurations than observed both in the FGFP (90% versus 12%; *n* = 10 versus 1,106, pairwise chi-squared test = 48.7, *P*_adj_ = 1.2 × 10^−11^) and the 4+ CGC (90% versus 22%; *n* = 10 versus *n* = 91, pairwise chi-squared test = 16.9, *P*_adj_ = 1.6 × 10^−4^; Fig. 1f and Supplementary Table 4).

### Exclusion of young children reveals familial patterns in microbiota variation

To characterize cross-generational familial microbiome similarity, we first assessed variation in abundance patterns of microbial taxa and functions within and between families. Given their diverging microbiomes (Fig. 1d,f), we opted to exclude participants younger than 4 from these analyses, leaving us with an *n* = 91 cohort (4+ CGC). Exclusion of young children resulted in family identifier being the sole significant microbiome covariate, accounting for 14.7% of genus-level compositional variation (*n* = 91, genus-level stepwise dbRDA, *P*_adj_ = 5.5 × 10^−3^; Supplementary Table 2 and Fig. 1c), exceeding the effects sizes of previously identified microbiome covariates^26^. Hence, we conclude that among women with a mature colon microbial ecosystem, family-bound phylogenetic microbiome community patterns can be identified over multiple generations. Remarkably, no such significant association with family was observed when assessing interindividual variation in abundance patterns of core microbial metabolic pathway modules^28^ (*n* = 91, single dbRDA, *P* = 0.23). These results are in accordance with the concept of a functionally redundant gut microbial ecosystem^28^: while taxonomic profiles can vary substantially between individuals and even over time, taxa encode an overlapping core functional potential, ensuring stable interactions with the human host^29^. Of note, ARG abundance profiles also did not differ significantly between families in the 4+ CGC dataset (*n* = 91, single dbRDA, *P* = 0.27). Next, we zoomed in on specific microbiome features rather than community-level variation, capitalizing on the availability of quantitative microbiome profiling (QMP)-based, metagenome-derived genus abundances. We found that seven genera occurred in higher abundances among members of specific families compared to the rest of the 4+ CGC cohort (*n* = 91, two-sided Wilcoxon rank-sum test, −log_10_(*P*) > 4.56; Fig. 1g and Supplementary Table 5). While most of those family-associated genera could be qualified as low abundant (mean abundance < 3 × 10^6^ cells per gram of faeces, within 20% of the taxa with the lowest mean abundances in the dataset), 2 families were enriched in *Pseudomonas* (mean abundance = 3.74 × 10^6^), an opportunistic pathogen^30^, and *Oxalobacter* (mean abundance = 3.74 × 10^6^), linked to kidney stone risk reduction^31^, respectively. As a complementary approach, we assessed whether prevalence (presence/absence) of species or functions appeared family-bound across 4+ CGC generations (non-random distribution in families across the cohort genealogy)^32^. None of the features evaluated (species, core functions and ARGs) were shared more frequently between related individuals than expected by chance in the cohort (*n* = 91, genealogical index of familiality (GIF), *P*_adj_ > 0.05; Supplementary Table 6).

### Family members share closely related bacterial genotypes

**Fig. 2:**
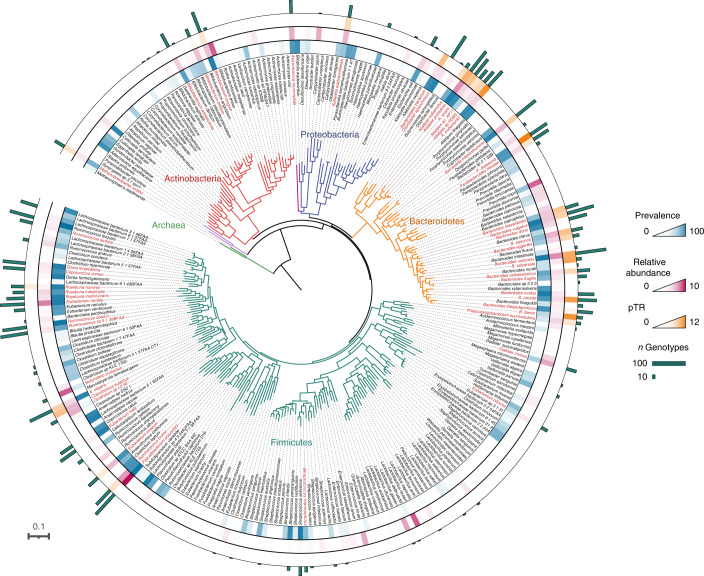
Maximum likelihood phylogenetic tree of the CGC species profiled in this study. All species detected within the CGC cohort and present in the PhyloPhlAn default database are shown; species profiled using StrainPhlAn are shown in red. Branches are coloured by phylum (Actinobacteria, red; Archaea, green; Bacteroidetes, yellow; Firmicutes, dark green; Proteobacteria, blue; Verrucomicrobia, pink), with the outer circles representing scaled prevalence (blue), relative abundance (percentage, pink) and pTRs (yellow). The number of genotypes recovered for each species is represented by bar plots (green).

**Fig. 3:**
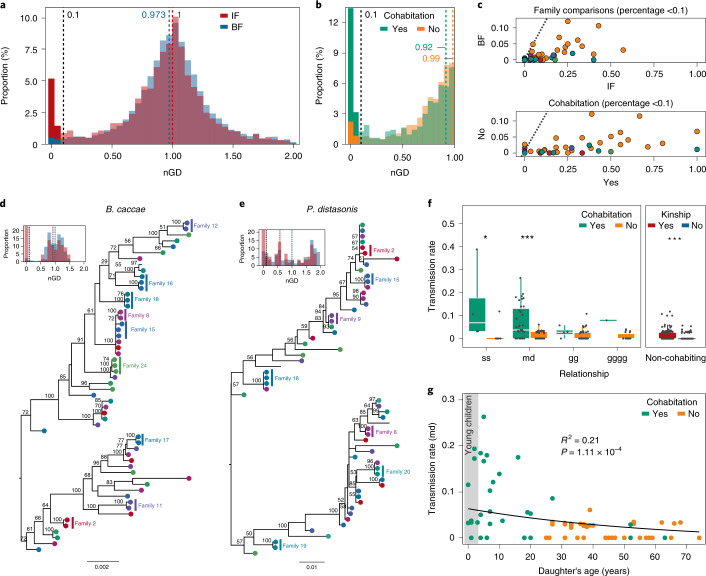
Transmissions of genotypes across family members. **a**, nGDs between all pairs of genotypes recovered. IF distances (red) present a lower overall median compared with BF distances (blue). **b**, nGDs for cohabiting individuals peak towards nGD = 0. **c**, Proportion of total pairwise comparisons with nGDs < 0.1. Top: Comparisons based on relatedness, with higher proportions of IF (*x* axis) compared with BF (*y* axis) for all taxa. Bottom: Cohabitation-based comparisons, with cohabiting (*x* axis) participants showing higher proportions compared to non-cohabiting (*y* axis) individuals. Taxa are coloured by phylum (Actinobacteria, red; Bacteroidetes, yellow; Euryarchaeota, green; Firmicutes, blue; Proteobacteria, violet; Verrucomicrobia, pink). The dashed line indicates 1:1 proportions. **d**,**e**, Maximum likelihood phylogenetic trees of species exhibiting the highest number of IF transmissions (Supplementary Table 13): *B. caccae* (**d**) and *P. distasonis* (**e**). Nodes are coloured by family ID (colour-coded as in Fig. 1a); family members that exchange strains are shaded. The vertical lines in the histograms indicate nGDs for IF (red) and BF (blue). **f**, Left: pTRs by relationship (ss, sister; md, mother–daughter; gg, grandmother–granddaughter; gggg, great-grandmother–great-granddaughter) and cohabitation status (green = yes, yellow = no) (Wilcoxon rank-sum test, *n* = 102, *P_adj_ < 0.05, ***P_adj_ < 0.001; Supplementary Table 15). Right: pTRs by kinship for non-cohabiting individuals (Wilcoxon rank-sum test, *n* = 94, *r* = 0.24, *P* = 5.85 × 10^−5^). The body of the box plot represents the first and third quartiles of the distribution and the median line. The whiskers extend from the quartiles to the last data point within 1.5× the IQR, with outliers beyond. **g**, The pTR between mothers and daughters decreases with the daughter’s age (*n* = 78 pairs, beta regression, *R*^2^ = 0.21, *z* = −3.87, *P* = 1.11 × 10^−4^).

**Extended Data Fig. 3:**
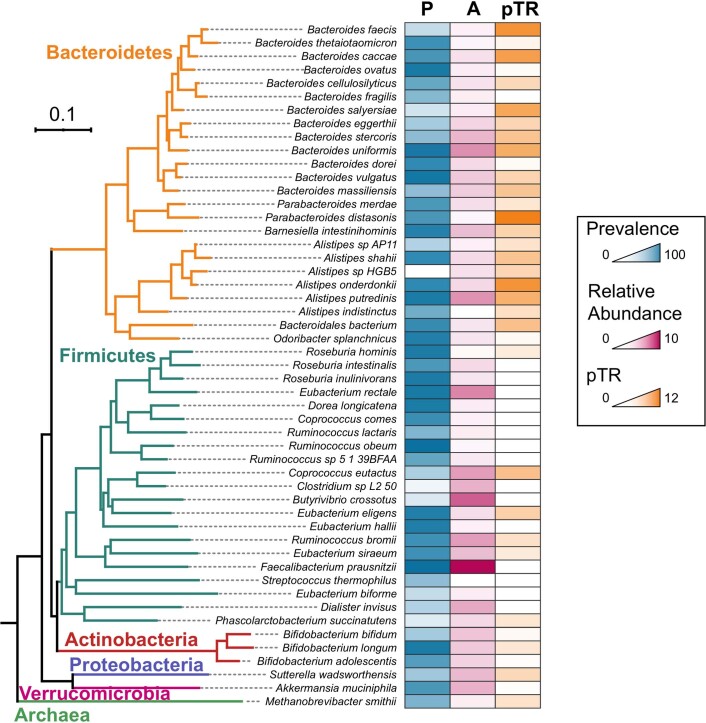
Maximum-likelihood phylogenetic tree of the species present within the CGC cohort analysed using StrainPhlAn. Branches are coloured by phyla (Actinobacteria, red; Archaea, green; Bacteroidetes, yellow; Firmicutes, dark green; Proteobacteria, blue; Verrucomicrobia, pink). Boxes represent % prevalence (P, blue), % relative abundance (A, pink), and potential transmission rates (pTR, yellow).

The detection of familial microbiome community patterns does not necessarily reflect actual transmission of microorganisms across generations but could also result from shared genetic backgrounds and cultural transmission of lifestyle and dietary habits selecting for a similar microbial composition^15,33^. To infer potential exchange or co-acquisition of microbial strains between members of the same family, we recovered representative genotypes (consensus genetic sequences resulting from concatenation of marker genes with complete coverage) of species present with sufficient coverage in the unrarefied CGC faecal shotgun metagenomes using StrainPhlAn. This approach allowed us to characterize over 360 species across the CGC dataset (including samples from young children, *n* = 102; Fig. 2 and Supplementary Table 7). Focusing on species detected at least 3 times within a single family and having a core genome alignment higher than 1,000 base pairs (bp), we restricted our analyses to 2,374 genotypes representing 51 species (median genotypes per species = 44, range = 13–92; Extended Data Fig. 3 and Supplementary Table 8), together constituting a substantial fraction of the CGC metagenomes (median = 77.04%, range = 7.35–91.85%; Supplementary Table 1). For each species, we calculated the genetic distances between all pairs of genotypes recovered as the number of single-nucleotide polymorphisms (SNPs) (Supplementary Table 9). Overall, for these 51 species, the normalized genetic distances (nGDs) (normalized by the median intraspecies genetic distance as proposed by Ferretti et al.^2^) between genotypes recovered from family members (intrafamily (IF) were lower than those observed between non-related individuals (between-family (BF)); median nGD_IF_ = 0.973 versus nGD_BF_ = 1; *n* = 102, permutational multivariate analysis of variance (PERMANOVA) on median nGDs, *R*^2^ = 0.304, *P* = 1 × 10^−3^; Fig. 3a), indicating that more similar strains could be found within than across families. Analysed per species, a similar pattern was observed for 13 out of the 51 taxa genotyped (PERMANOVA, *P*_adj_ < 0.05; Supplementary Table 10). Of note, the overall distribution of IF distances showed a peak at nGD = 0 (that is, identical strains) whereas the BF one did not, suggesting a higher frequency of person-to-person transmissions and/or recent acquisition of microorganisms from a common source^34^. Estimating the proportion of genotype pairs falling within this nGD = 0 peak by fitting a Gaussian mixture model, we confirmed the fraction of high-similarity pairs to be significantly higher between related participants than non-family members (IF = 5.71% versus BF = 2.06%; *n*_IF_ = 2,450 versus *n*_BF_ = 63,287, two-proportion test, chi-squared = 86.848, *P* < 2.2 × 10^−16^; Fig. 3a). Similarly, family members sharing a household cohabitation presented a significantly higher proportion of closely related genotypes compared to those living apart (LA) (cohabitation = 14.27% versus LA = 1.81%; *n*_cohabitation_ = 633 versus *n*_LA_ = 1,817, two-proportion test, chi-squared = 28.857, *P* = 7.79 × 10^−8^; Fig. 3b). This finding aligns with the hypothesis of a higher probability of transmission or co-acquisition of gut microbes among household members due to the closeness and frequency of their contacts^33,35^. Both within family and household, highly similar genotypes primarily belonged to the phylum Bacteroidetes (Fig. 3c and Supplementary Table 10). Applying a similar approach on ARGs, we additionally computed all pairwise genetic distances between ARG sequences retrieved from CGC individuals (*n* = 533 ARG clusters). Evaluating the distribution of nGDs between ARG variants within and between families and among family members living together or apart, the differences observed (uncorrected for multiple testing; PERMANOVA, *P* < 0.05; Supplementary Table 11) corresponded to more closely related sequences shared by family members (12.31%, *n* = 64 out of 520) and participants living together (15.13%, *n* = 59 out of 390).

### Bacteroidales species display the highest potential transmission rates

**Extended Data Fig. 4:**
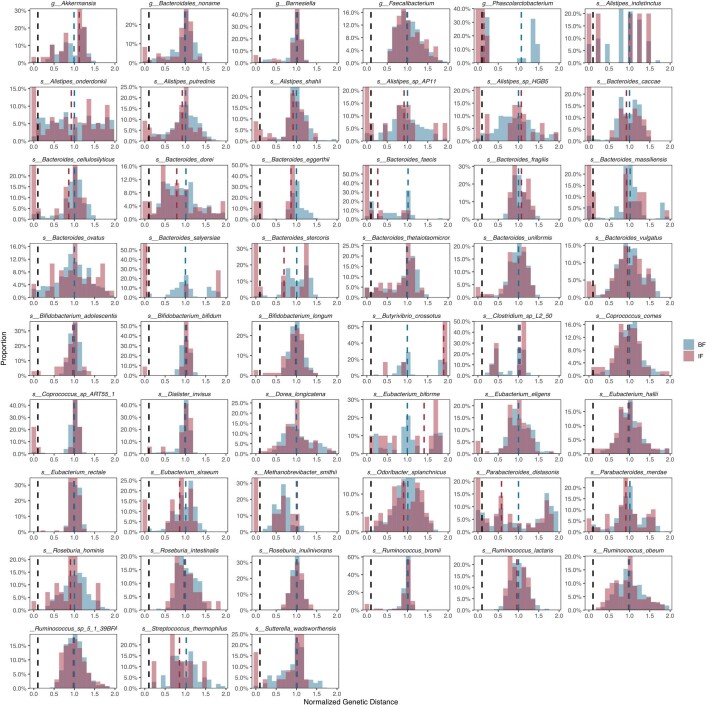
Distribution of normalized genetic distances for each of the 51 species analysed. Distances between pairs of genotypes recovered from individuals of the same families are coloured in red, and those from individuals of different families are coloured in blue. Dashed vertical lines represent the threshold used to define two genotypes belong to the same strain (nGD < 0.10; in black), intra-family median distances (red) and between-family median distances (blue).

**Extended Data Fig. 5:**
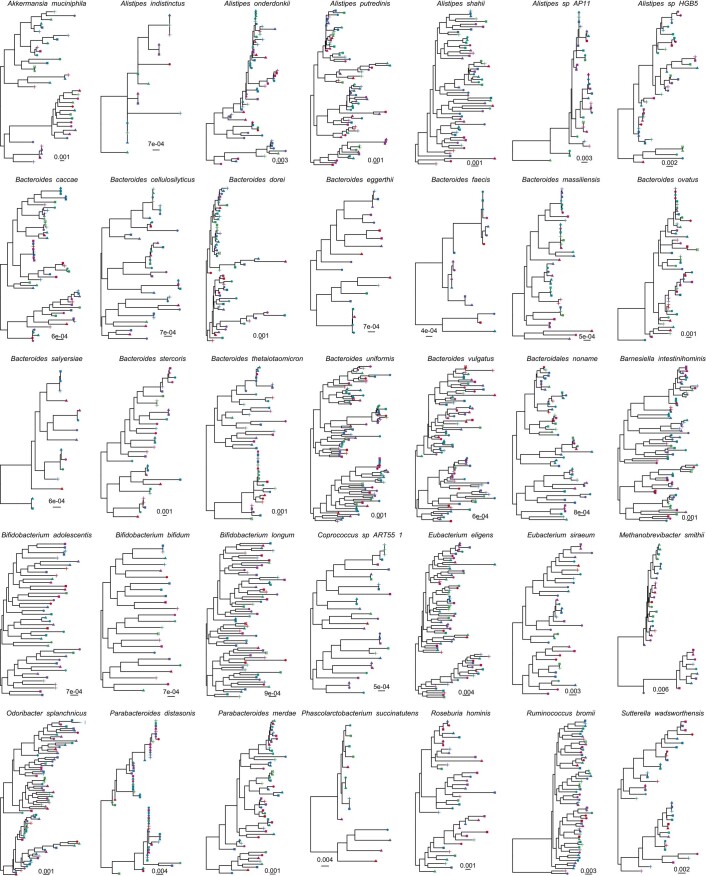
Phylogenetic trees of species for which strain sharing was detected between at least two members of the same family. Tips are colored by family IDs as in Fig. 1 and shapes indicate generation number (0 = Circle, 1=Triangle, 2=Square, 3=Cross).

While sharing of strains can result from co-acquisition of bacterial species, increased frequencies of shared strains have been suggested to be indicative for transmission between individuals^36^. In this study, based on the observed distribution of nGDs and opting for the most stringent among previously suggested similarity thresholds^2,5,37^, we considered two genotypes to belong to the same strain when their nGDs < 0.10 (Fig. 3a and Extended Data Fig. 4). This cut-off allowed us to identify 1,958 strains in the CGC cohort, with 213 of them (belonging to 40 species) being involved in a total of 870 potential transmission events (Supplementary Table 9 and Supplementary Table 10). Such events were observed to occur significantly less frequently among unrelated individuals compared to family members (IF = 42.02% versus BF = 12.87%; *n*_IF_ = 188 versus *n*_BF_ = 4,912 pairs of individuals, chi-squared test = 125.87, *P* < 2.2 × 10^−16^). Potential intrafamilial transmission events were detected for 35 out of the 51 species genotyped, together representing more than 80% of the dominant genera in the gut microbiota (defined as the top 20% most abundant genera). To quantitatively explore these observations, we calculated potential transmission rates (pTRs) within as well as across species as the number of transmission/co-acquisition events detected divided by the maximum possible transmissions in a family (defined as the combinations of family members: maximum = *nCr*(*n* = *n* members, *r* = 2)). Across species, average pTRs varied substantially between families (mean = 2.75%, range 0–9.85%; Supplementary Table 13), putatively reflecting differences in familial interaction patterns or habits such as hygiene practices^38^. Within species, we observed the highest average pTRs for members of the order Bacteroidales (including *Parabacteroides distasonis* = 11.11% (0–50%), *Alistipes onderdonkii* = 9.58% (0–100%), *Bacteroides faecis* = 8.75% (0–50%), *Bacteroides caccae* = 8.61% (0–50%) and *Bacteroides salyersiae* = 8.33% (0–50%); Fig. 2 and Supplementary Table 13), in line with reports on their frequent transmission from mother to offspring^5^. To visualize IF strain sharing across the CGC dataset, we constructed a maximum likelihood phylogenetic tree based on the genotypes recovered within the cohort for each of the 35 species potentially transmitted between family members (Fig. 3d,e, Extended Data Fig. 5 and Supplementary Table 13). The highest numbers of potential IF transmission/co-acquisition events were detected for *B. caccae* and *P. distasonis*, shared between 15 and 14 pairs of individuals within 9 and 10 families, respectively (Fig. 3d,e).

### Both kinship and cohabitation are associated with higher potential strain transmission

**Extended Data Fig. 6:**
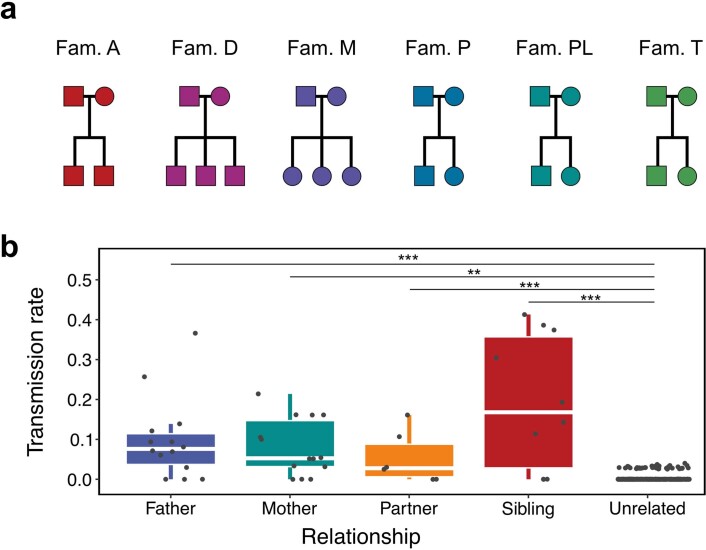
Summary results for cohabitating, non-related individuals. (**a**) Family structures in the Costea *et al*. study (N = 26)^39^. (**b**) pTRs by relationship (KW, N = 26, Chi2 = 105.65, P < 2.2e-16; PhD tests for IF adjP>0.05; IF groups vs unrelated adjP<0.001(***), <0.01(**); Supplementary Table 18). The body of the box plot represents the first and third Quartiles of the distribution and the median line. The whiskers extend from the quartiles to the last data point within 1.5× the interquartile range, with outliers beyond.

In the present dataset, kinship, cohabitation status and even age—all potential covariates of bacterial transmission frequency—emerged as closely related variables. For instance, 94% of participants under 30 years old reported living together with their mothers, with 96.3% of *n* > 2 households comprising a least 1 mother–<30-year-old daughter pair (Supplementary Table 1). While strain distribution was significantly associated with kinship (strain presence/absence microbiome profile variation, *n* = 102, Mantel test, *R*^2^ = 3.7%, *P* = 6.5 × 10^−3^), only cohabitation had a significant non-overlapping effect size (stepwise RDA, *R*^2^ = 8.1%, *P*_adj_ = 1.3 × 10^−3^; Supplementary Table 14). Within families, the highest pTRs were observed within sister (*n* = 13 pairs, mean = 5.17% (0–38.71%)) and mother–daughter pairs (*n* = 78, mean = 3.99% (0–26.32%)). While pTRs spanning multiple generations were markedly lower (*n* = 49, mean = 1.33% (0–10.71%) and *n* = 19, mean = 1.27% (0–7.9%) for pairs separated by 1 and 2 generations, respectively), only the differences between two (mother–daughter) and three generations (grandmother–granddaughter) were identified as significant within the limitations of our cohort (*n* = 102, Kruskal–Wallis test, chi-squared = 10.99, *P* = 1.18 × 10^−2^; post-hoc Dunn test, *P*_adj_ = 1.36 × 10^−2^; Supplementary Table 15). Both for sisters and mother–daughter pairs, the pTRs calculated between pairs of individuals cohabiting were significantly higher than among their counterparts living apart (Wilcoxon rank-sum test, sisters, *r* = 0.73, *P*_adj_ = 1.64 × 10^−2^; mother–daughter pairs, *r* = 0.47, *P*_adj_ = 1.56 × 10^−4^; Fig. 3f and Supplementary Table 15), again indicative of cohabitation potentially promoting exchange of gut bacteria. However, overall, the pTRs for pairs of non-cohabiting family members was higher compared to non-related individuals (Wilcoxon rank-sum test, *r* = 0.24, *P* = 5.85 × 10^−5^; Fig. 3f and Supplementary Table 15). To gain a better understanding of the impact of cohabitation on strain sharing or potential transmission events, we reanalysed a family cohort assembled by Costea et al.^39^ consisting of 26 individuals belonging to 6 households (parents and offspring; Extended Data Fig. 6a). Applying the methodology described above, 43 species covering 498 strains were considered eligible for pTR analysis (Supplementary Table 17). Distinguishing between strains being shared among cohabiting related individuals (mother/father–offspring, *n* pairs = 28) and between partners (father–mother, *n* pairs = 6), we found that both categories exhibited higher pTRs than non-related, non-cohabiting individuals (*n* = 26, Kruskal–Wallis test, chi-squared = 105.65, *P* < 2.2 × 10^−16^; post-hoc Dunn test, *P*_adj_ < 0.01; Extended Data Fig. 6b and Supplementary Table 18), albeit with smaller effect and sample sizes for partners. Hence, while our analyses identified kinship as a key covariate of genus-level microbiota community differentiation, both CGC and the Costea et al.^39^ (re)analyses do not exclude cohabitation to be the driving factor in transmission or co-acquisition of individual microbiome features, which is in line with the findings of recent studies on gut ecosystem heritability^40,41^.

**Extended Data Fig. 7:**
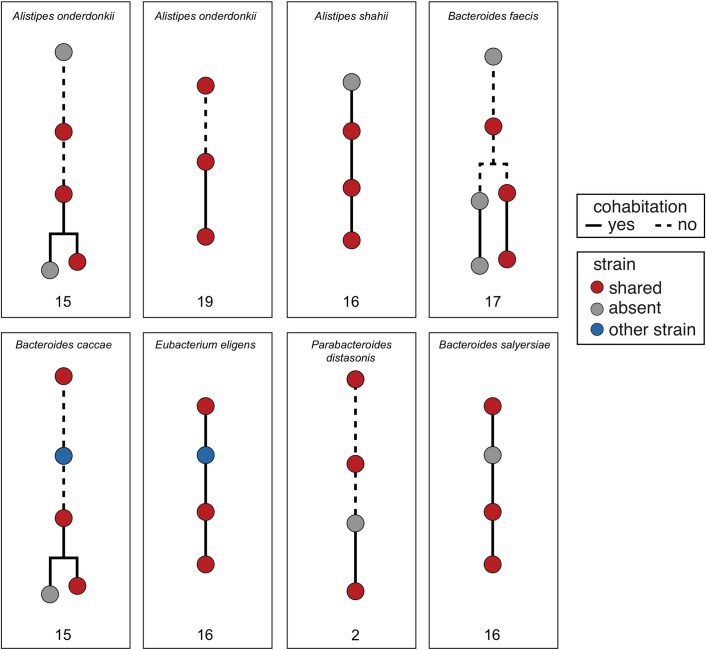
Strains shared across three consecutive generations (top) and across four generations but missing one intermediate level (bottom). Numbers indicate family IDs for which strain sharing over more than two generations was observed.

Potentially reflecting the physical intimacy of their relation, we detected the highest average pTRs between mothers and daughters in pairs comprising younger children, with frequencies steadily decreasing with age (*n* = 78 pairs, beta regression, *R*^2^ = 0.21, *z* = −3.87, *P* = 1.11 × 10^−4^; Fig. 3g)—an association again clearly linked to cohabitation, although the addition of this parameter did not significantly improve the correlation (*R*^2^ = 0.24, model comparison likelihood ratio test *P* = 0.16). Also, among the species shared between mothers and young children, the largest pTRs were observed for Bacteroidetes, notably *B. caccae* (mean = 57.14%), *Bacteroides stercoris* (mean = 33.33%) and *P. distasonis* (mean = 28.57%; Supplementary Table 16). Although our analyses did not allow to resolve directionality, with pTRs also reflecting potential transmission from daughters to mothers^42^, our findings do not contradict the hypothesis of the maternal gut ecosystem being a contributor to primary succession events that constitute microbiota maturation processes in young children^2,19^. In this respect, given its low colonization resistance^43^, the immature nature of the infant and toddler microbiota can be expected to facilitate inclusion of exogenous microbiome features, acquired through both vertical and horizontal transmission or originating from environmental sources. Finally, we observed four strains belonging to the species *A. onderdonkii* (two strains), *Alistipes shahii* and *B. faecis* to be present in three consecutive generations in four families, potentially reflecting persistent niche colonization across generations (Extended Data Fig. 7). In addition, the strains of four other species were detected across three non-consecutive generations in three families. *B. salyersiae* and *P. distasonis* remained undetected in one of the intermediate levels, while a different strain of *B. caccae* and *Eubacterium eligens* were found at the grandmother level (Extended Data Fig. 7).

## Conclusion

Our explorative analyses of gut microbiota variation across generations confirmed the microbiome of young children to be fundamentally divergent from more developed configurations, with familial community structures only emerging on ecosystem maturation. Although the impact of kinship was additionally reflected in a higher frequency of strain sharing between family members compared to unrelated individuals, estimations of pTRs identified cohabitation as a key covariate of strain distribution. In line with these findings, we observed IF pTRs to decrease both with degree of kinship and age difference, with potential transmission events across generations being rare but detectable. Shared strains predominantly belonged to the Bacteroidales order. Overall, while our analysis does not exclude cross-generational transmission of strains resulting from maternal inheritance, strain sharing was most frequently detected among first-degree relatives sharing a household.

## Methods

### Ethical compliance

All experimental protocols were approved by the Medical Ethics Committee Universitair Ziekenhuis Brussel-Vrije Universiteit Brussel (BUN 143201215505) and the Commissie voor Medische Ethiek, Universitair Ziekenhuis/Katholieke Universiteit Leuven (S58125). Study design complied with all relevant ethical regulations, aligning with the Declaration of Helsinki (2013 version) and in accordance with Belgian privacy legislation. Written informed consent was obtained from all adult participants and from the parents of underage participants. Participants did not receive compensation for their participation in the study.

### Sample collection

The cohort included 102 female participants belonging to families with at least 3 generations of women (*n* = 24 families, median = 4 generations per family). Sampling took place between November 2015 and November 2016 and all participants signed a statement of informed consent. A limited set of data, including participant’s birth date, height, weight, delivery mode, antibiotic use over the last months and family structure was collected at enrolment (Supplementary Table 1). Faecal sample collection and blood analyses were performed as in Falony et al.^26^. Briefly, participants were asked to collect their faecal material (single defecation) in a plastic vial, place the vial in a labelled non-transparent ziplock bag and freeze it at −20 °C immediately after collection. Frozen samples were transported within 72 h to the research facility and stored at −80 °C. Blood samples were drawn by a study nurse and analysed by an independent certified clinical laboratory (Centrum voor Medische Analyse, Belgium). Participants were asked to refrain from calorie intake for 8 h before blood sampling.

### Statistics and reproducibility

While no statistical method was used to predetermine sample sizes for the present explorative study, CGC cohort size was similar to the number of participants included in previous publications^2,39^. Data exclusions are specified and justified for each of the analyses presented. Experiments were not randomized in this explorative cross-sectional study but the Costea et al.^39^ dataset was used to replicate findings. No intervention was performed on participants; thus, they were not randomly allocated into study groups. Data collection and analysis were not performed blinded to the conditions of the study set-up.

### Faecal sample characterization

**Extended Data Fig. 8:**
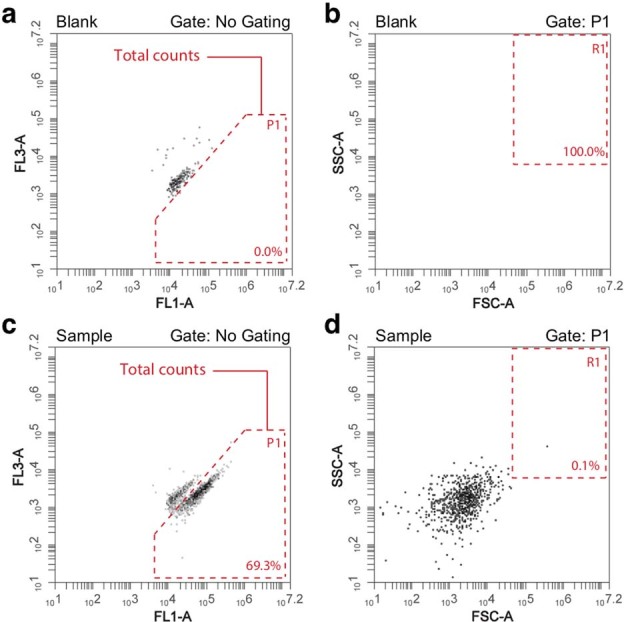
Illustration of flow cytometry gating strategy. A fixed gating/staining approach was applied. Both blank and sample solutions were stained with SYBR Green I. (**a**) FL1-A/FL3-A acquisition plot of a blank sample (0.85% w/v physiological solution) with gate boundaries indicated. A threshold value of 2000 was applied on the FL1 channel. (**b**) Secondary gating was performed on the FSC-A/SSC-A channels to further discriminate between debris/background and microbial events. (**c**, **d**) FL1-A/FL3-A count acquisition of a faecal sample with secondary gating on FSC-A/SSC-A channels based on blank analyses. Total counts were defined as events registered in the FL1-A/FL3-A gating area excluding debris/background events observed in the FSC-A/SSC-A R1 gate. The flow rate was set at 14 microliters per minute and the acquisition rate did not exceed 10,000 events per second. Each panel reflects the events registered during a 30 s acquisition period. Cell counts were determined in duplicate starting from a single biological sample.

To assess microbial loads in faecal samples, 0.2 g frozen (−80 °C) aliquots were diluted 100,000 times in physiological solution (8.5 g l^−1^ NaCl; VWR International). Samples were filtered using a sterile syringe filter (5 µm pore size; Sartorius Stedim Biotech) and 1 ml of the resulting microbial cell suspension was stained with 1 µl of SYBR Green I (1:100 dilution in dimethyl sulfoxide; shaded 15 min incubation at 37 °C; 10,000 concentrate; Thermo Fisher Scientific). Microbial cell count (*n* = 101; Supplementary Table 1) was performed using an Accuri C6 flow cytometer (BD Biosciences) based on Prest et al.^44^. Fluorescence events were recorded using the FL1 533/30 nm and FL3 > 670 nm optical detectors; forward and sideward scattered light signals were collected. The BD Accuri CFlow software v.1.0.264.21 was used to gate and separate the microbial fluorescence events on the FL1/FL3 density plot from the faecal sample background. A threshold value of 2,000 was applied to the FL1 channel. To exclude any remaining background events, gated fluorescence events were evaluated on the forward/sideward density plot. Instrument and gating settings were kept identical for all samples (fixed staining/gating strategy^44^; Extended Data Fig. 8). Cell counts were converted to microbial loads per gram of faecal material based on the exact weight of the aliquots analysed. Measurements were performed in duplicate; if the number of events recorded differed by more than 10%, a third replicate was measured. One sample was excluded from cell counting due to insufficient faecal material to perform the measurements. Moisture content was determined as the percentage of mass loss after lyophilization from approximately 0.2 g frozen aliquots of faecal material (−80 °C). Faecal calprotectin concentrations were determined using the fCAL ELISA Kit (Bühlmann) on frozen faecal material (−80 °C).

### DNA extraction, sequencing and data preprocessing

Faecal DNA extraction and microbiota profiling was performed as described previously^45^. Briefly, DNA was extracted from faecal material using the MoBio PowerMicrobiome RNA Isolation Kit, with the addition of 10 min incubation at 90 °C after the initial vortexing step.

For amplicon sequencing, the V4 region of the 16S rRNA gene was amplified with the primer pair 515F/806R^46^. Sequencing was performed on the Illumina MiSeq platform to generate paired-end reads of 250 bases in length in each direction. 16S data preprocessing was performed using LotuS^47^ v.1.565 to demultiplex the sequencing reads. Amplicon sequencing was used only for community typing to align with the FGFP dataset.

Whole-metagenome shotgun sequencing was performed using the Illumina HiSeq 2500 system (151 bp paired-end reads; Novogene). Paired-end reads were first quality-checked using fastqc v.0.11.2 and Illumina adaptors and low-quality reads were removed using Trimmomatic^48^ v.0.32 with the options ILLUMINACLIP:trimmomatic-0.32/adapters/NexteraPE-PE.fa:2:30:10:2, MAXINFO:40:0.70, HEADCROP:15 and MINLEN:40. High-quality reads were then decontaminated from phiX and human sequences using DeconSeq^49^ v.0.4.3 and broken pairs of reads (pairs for which one member was removed during filtering) were identified and removed using a custom script, available at https://github.com/raeslab/raeslab-utils/.

### Relative and quantitative microbiome taxonomic profiling

Taxonomical assignment of preprocessed 16S data was performed using the DADA2^50^ pipeline v.1.6.0 and the RDP classifier^51^ v.2.12 with default parameters. To obtain the 16S relative microbiome profiling (RMP) matrix, each sample was downsized to 10,000 reads by random selection of reads. Samples with less than 10,000 reads were excluded (1 sample) from the analyses.

Using sequencing data decontaminated from phiX and human sequences to generate the shotgun QMP matrix, shotgun sampling size was defined as the average abundance of ten universal single-copy marker genes of the MOCAT2^52^ pipeline (COG0012, COG0016, COG0018, COG0172, COG0215, COG0495, COG0525, COG0533, COG0541, COG0552). Paired-end reads were downsized to even sampling depth (ratio between sampling size and microbial load, that is, the average total cell count per gram of frozen faecal material) by random selection of the reads to equate the minimum observed sampling depth in the dataset (minimum sampling depth = 4.98 × 10^−9^). The resulting rarefied read counts were above 1.3 × 10^6^ reads for all samples. Next, taxonomic classification of the rarefied reads into molecular operational taxonomic units (mOTUs) was performed with MOCAT2^52^ v.2.0.1 based on the abundances of the single-copy marker genes, with default parameters and skipping any filtering or trimming steps. mOTUs were then aggregated into species and genera using mOTU taxonomic annotation (mOTU.v1 database). Microbiome profiles were converted to the numbers of cells per gram by dividing by the total mOTU linkage group abundance in the sample (including mOTUs with no phylogenetic assignment) and multiplying by the number of cells per gram of faeces. In addition, taxonomic profiling at the species and strain levels were performed using MetaPhlAn2^53^ and StrainPhlAn2^36^. Briefly, the preprocessed metagenomic reads were mapped against the MetaPhlAn2 marker database using the metaphlan2 script with default parameters. Then, samples2markers.py was run to produce the gene marker file for each sample; gene marker files were parsed to StrainPhlAn2 to identify the taxa detected in each metagenomic sample. Rarefied abundances at the genus, species and strain levels were also converted into number of cells per gram as described for the mOTUs.

### Quantitative microbiome functional profiling

QMP-rarefied reads were mapped on the integrated gene catalogue (IGC)^54^ using the Burrows–Wheeler Aligner^55^ v.0.7.8 and the mapping was summarized into functional profiles by featureCounts^56^ v.1.5.3, with the parameters --minOverlap 40 --pO). Gut metabolic module (GMM)^28^ abundances were computed using Omixer-RPM v.1.0 (https://github.com/raeslab/omixer-rpm), with option -c 0.66 (66% coverage detection threshold). Coverage of the manually curated modules is calculated as the number of pathway steps for which at least one of the orthologous groups is found in a metagenome, divided by the total number of steps constituting the module. The rarefied reads mapped on the IGC were also annotated with ARGs using the Comprehensive Antibiotic Resistance Database^57^. GMM and ARG abundances were converted to quantitative abundance profiles (abundance per gram of faeces) by dividing by total mOTU linkage group abundance in the sample (including mOTUs with no phylogenetic assignment) and multiplying by the number of cells per gram of faeces.

### Identification of species-representative genotypes

To identify the species genotypes in the dataset, we used StrainPhlAn^36^ on the original, non-rarefied reads to produce covered core alignments of marker genes as indicated above. As such, the consensus genetic sequence resulting from the concatenation of marker genes for each species and individual is referred to as genotype. Taxonomic groups corresponding with phages, viruses and viroids were discarded from further analysis. Gaps were removed from the alignments using T-Coffee^58^ v.11.00 with option -action +rm_gap 1 so that only the covered core genome for that particular comparison was analysed; *SNP-sites*^59^ v.2.5.1 was used to obtain the alignments of SNPs. Only alignments that contained 3 or more samples from at least 1 family and core genome sizes of 1,000 bp were kept.

### Genetic distances and phylogenetic analysis

Core genome alignments were used to compute the pairwise genetic distances between all genotypes of each species by using snp-dists v.0.6 (https://github.com/tseemann/snp-dists). The genetic distances, calculated as the number of SNPs between pairs of genotypes, were divided by the length of the core genomes to obtain the number of SNPs per megabase. In addition, distances were normalized by the median genetic distance of each taxa (nGDs). We considered that two genotypes belonged to the same strain if their nGD was below the stringent threshold of 0.10, as used by others^2,5^. To reconstruct the phylogenetic trees from the previously obtained core genome alignments, we used RAxML v.8.2.12^60^ with the parameters -f a and -m GTRGAMMA. For the phylogenetic trees obtained with the strainphlan.py script, we set bootstrap_raxml to 100 and marker_in_clade to 0.2. Phylogenetic trees were rooted midpoint with the package ETE 3^61^. Finally, PhyloPhlAn v.3.0.60^62^ was used to produce a phylogenetic tree of all the species profiled using MetaPhlAn2 and the associated metadata were plotted using iTOL v6^63^. For the 51 species analysed at the strain level, we selected proximal representatives of taxa absent in the PhyloPhlAn database.

### Antimicrobial resistance genes

The presence of sequence-identical antimicrobial resistance genes (ARGs) across individuals was assessed by extracting consensus sequences corresponding with ARGs from the IGC alignment^64^, filtering by gene length coverage above 99% and 5 reads of minimum depth. Next, for each gene, we computed the pairwise genetic distances between pairs of individuals, as described for genotypes.

### Statistical analyses

Statistical analyses were performed in R using the packages vegan^65^ v.2.5.6, phyloseq^66^ v.1.32.0, FSA^67^ v.0.8.30, coin^68^ v.1.3.1, DirichletMultinomial^69^ v.1.30.0, kinship2 (ref. ^70^) v.1.8.5, FamAgg^71^ v.1.16.0, QuantPsyc^72^ v.1.5, gmm^73^ v.1.6.5 and ggplot2 (ref. ^74^) v.3.3.2. Non-parametric statistical tests were used because data did not follow normality or equal variance assumptions. All *P* values were corrected for multiple testing using the Benjamini–Hochberg method (reported as *P*_adj_) unless specified otherwise and significance was defined as *P* < 0.05 and *P*_adj_ < 0.05.

#### Microbiota community variation explained by metadata variables

Contribution of metadata variables (age, SBMI, delivery mode, family ID, cohabitation status, medication use, antibiotic use, moisture content (%) and faecal calprotectin (μg g^−1^)) to interindividual microbiota community variation was determined by single dbRDA on genus-level Bray–Curtis dissimilarity with the capscale function in the vegan R package^75^. The cumulative contribution of metadata variables was determined by forward model selection on dbRDA with the ordiR2step function in vegan, with variables that showed a significant contribution to microbiota community variation (P_adj_ < 0.05) in the previous step.

#### Faecal microbiome-derived features and visualization

Observed genus richness was calculated on the QMP matrix using phyloseq^66^. Enterotyping (or community typing) based on the DMM approach was performed in R using the DirichletMultinomial^69^ package as described by Holmes et al.^25^ on the RMP matrix. To increase accuracy, enterotyping was performed on a combined genus abundance matrix including the present dataset (*n* = 101) complemented with 1,106 samples from the FGFP^26^ cohort rarefied to 10,000 reads. Microbiome interindividual variation was visualized by principal coordinate analysis (PCoA) using Bray–Curtis dissimilarity on the genus-level abundance matrix. The optimal number of Dirichlet components based on the Bayesian information criterion was four. The four FGFP clusters were named *Prevotella, Bacteroides* 1, *Bacteroides* 2 and *Ruminococcaceae* as described by Vandeputte et al.^14^. The first has high relative abundance of *Prevotella* and the fourth has the highest genus-level richness, while the other two are dominated by the *Bacteroides* genus, with *Bacteroides* 2 also harbouring reduced *Faecalibacterium* abundance.

#### Microbiome and metadata associations

Taxa unclassified at the genus level or present in less than 10% of samples were excluded from the statistical analyses. Spearman correlations were used for rank-order correlations between continuous variables, including genera abundances, microbial loads, CRP and age. Wilcoxon rank-sum tests were used to test the differences of continuous variables between two different groups. For more than two groups, Kruskal–Wallis tests with post-hoc Dunn tests were applied. Statistical differences in the proportions of categorical variables (enterotypes) among groups were evaluated using pairwise chi-squared tests.

#### Microbiome transmission

Pedigrees were built using the kinship2^70^ R package. The GIF was calculated using the genealogicalIndexTest function (FamAgg^71^ package) to assess family aggregation of specific microbiota traits across the kinship matrix; binomial tests (binomialTest function) were used to test for enrichment of specific traits in certain families.

#### Analyses of genetic distances

nGDs between pairs of genotypes recovered from individuals were annotated by familial relationship and current cohabitation status as follows: IF, BF, cohabitation and LA. Comparisons between the nGD distributions between groups (family and cohabitation status) were performed using the generalized method and two-proportions test in the gmm R package. Wilcoxon rank-sum tests were used to test the median differences of the nGDs between two groups (BF versus IF). We also used PERMANOVA (adonis.test function in vegan*)* to test for differences between BF and IF. A similar approach was applied to evaluate the effects of kinship and cohabitation on the pairwise genetic distances between ARG sequences.

#### Identification of strains and calculation of pTRs

The nGDs between pairs of genotypes were used to define strains by grouping the pairs of genotypes from the same species with nGDs below 0.10. Transmission events between pairs of individuals were computed by adding up all species comparisons with nGD < 0.10 between any pair of individuals. pTRs between pairs of individuals were calculated by dividing the number of transmissions identified by the maximum possible transmissions in the pair (*n* shared strains per *n* species detected in any of the two individuals). The IF pTRs for each species were obtained by dividing the number of transmission events identified in a family for a certain species by the maximum possible transmissions in a family (maximum = *nCr* (*n* = *n* family members, *r* = 2)).

#### Analyses of pTRs

Statistical differences in the counts of transmissions among groups were evaluated using pairwise chi-squared tests. Spearman correlation analyses were performed to identify correlations between pTRs and continuous variables, including family size, carriers and prevalence of species in the population. To model the pTRs between mother and daughter pairs in relation to age, a generalized regression with beta response distribution (for response variables bound between 0 and 1) was fitted by maximum likelihood (betareg function in the betareg R package^76^,v3.1-4). The pTRs, with range = 0–1, were transformed to obtain rates in the range = 0–1 as pTR = (pTR(*n* − 1) + s)/*n*, with s = 0.5 as recommended for beta regression^77^. Nested model comparison was performed using a likelihood ratio test (lrtest in the lmtest R package^78^ v.0.9.38). For comparisons of pTRs between different types of kinship (sister, mother–daughter, grandmother–granddaugther), a Wilcoxon rank-sum test was used if only two groups were compared; a Kruskal–Wallis test with post-hoc Dunn test was used if more than two groups were compared.

### Reporting Summary

Further information on research design is available in the Nature Research Reporting Summary linked to this article.

## Supplementary information

Reporting Summary

Supplementary Table Excel file containing Supplementary Tables 1–18.

## Data Availability

The raw amplicon sequencing data and shotgun metagenomics sequencing data reported in this study have been deposited in the European Genome-phenome Archive under accession nos. EGAS00001005651 and EGAS00001005649.

## Code availability

The custom script used to identify and remove broken pairs of reads (pairs for which one member was removed during filtering) is available at https://github.com/raeslab/raeslab-utils/.
