# Supplementary material for: Variation and transmission of the human gut microbiota across multiple familial generations
Source: Nat Microbiol. 2021 Dec 30;7(1):87–96. doi: 10.1038/s41564-021-01021-8 (PMC8727295; doi:10.1038/s41564-021-01021-8)
Supplement: Supplementary file 1 — Reporting Summary [file 41564_2021_1021_MOESM1_ESM.pdf]

nature research

Corresponding author(s): Jeroen Raes

Last updated by author(s): Nov 5, 2021

Reporting Summary

Nature Research wishes to improve the reproducibility of the work that we publish. This form provides structure for consistency and transparency in reporting. For further information on Nature Research policies, see our Editorial Policies and the Editorial Policy Checklist.

Statistics

For all statistical analyses, confirm that the following items are present in the figure legend, table legend, main text, or Methods section.

n/a	Confirmed	
☐	☒	The exact sample size (*n*) for each experimental group/condition, given as a discrete number and unit of measurement
☐	☒	A statement on whether measurements were taken from distinct samples or whether the same sample was measured repeatedly
☐	☒	The statistical test(s) used AND whether they are one- or two-sided<br>*Only common tests should be described solely by name; describe more complex techniques in the Methods section.*
☐	☒	A description of all covariates tested
☐	☒	A description of any assumptions or corrections, such as tests of normality and adjustment for multiple comparisons
☐	☒	A full description of the statistical parameters including central tendency (e.g. means) or other basic estimates (e.g. regression coefficient) AND variation (e.g. standard deviation) or associated estimates of uncertainty (e.g. confidence intervals)
☐	☒	For null hypothesis testing, the test statistic (e.g. *F*, *t*, *r*) with confidence intervals, effect sizes, degrees of freedom and *P* value noted<br>*Give P values as exact values whenever suitable.*
☒	☐	For Bayesian analysis, information on the choice of priors and Markov chain Monte Carlo settings
☐	☒	For hierarchical and complex designs, identification of the appropriate level for tests and full reporting of outcomes
☐	☒	Estimates of effect sizes (e.g. Cohen's *d*, Pearson's *r*), indicating how they were calculated

Our web collection on statistics for biologists contains articles on many of the points above.

Software and code

Policy information about availability of computer code

Data collection	No software was used for microbiome data collection. To assess faecal microbial loads, flow cytometry analysis was performed using the BD Accuri CFlow software (v1.0.264.21) for gating and event counting.

Data analysis	16S data pre-processing was performed using LotuS (version 1.565, used for demultiplexing sequencing reads) and the DADA2 pipeline (version 1.6.0), with the RDP classifier (version 2.12) for taxonomy assignment.<br><br>For analysis of shotgun sequencing data, paired-end reads were quality checked using fastqc (version 0.11.2), and Illumina adapters and low-quality reads trimmed with Trimmomatic (version 0.32), decontaminated from phiX and human sequences using DeconSeq (version 0.4.3), and broken pairs were fixed using a custom Biopython script (available at https://github.com/raeslab/raeslab-utils/).<br>Taxonomic classification of the rarefied reads into mOTUs was performed with MOCAT2 (version 2.0.1). Taxonomic profiling at the species and strain levels were performed using MetaPhlAn2 and StrainPhlAn2. Core alignments were computed by removing gaps using T-Coffee v11.00, and SNP-sites v2.5.1 was used to obtain SNP alignments. Pairwise genetic distances between all genotypes of each species were computed with snp-dists v0.6. Phylogenetic trees were computed with RAxML v8.2.12.<br><br>For functional profiling, QMP-rarefied reads were mapped on the integrated gene catalogue (IGC) using BWA (version 0.7.8), and the mapping was summarized into functional profiles by featureCounts (version 1.5.3, with parameters --minOverlap 40 –pO). GMM (gut metabolic module) abundances were computed using Omixer-RPM v1.0 (https://github.com/raeslab/omixer-rpm).<br><br>Data analysis and graphical representations were performed using R, a free software environment for statistical computing, with packages vegan (v2.5.6), phyloseq (v1.32.0), FSA (v0.8.30), coin (v1.3.1), DirichletMultinomial (v1.30.0), kinship2 (v1.8.5), FamAgg (v1.16.0), QuantPsyc (v1.5), gmm (v1.6.5), ggplot2 (v3.3.2), and lmtest (v0.9.38).

For manuscripts utilizing custom algorithms or software that are central to the research but not yet described in published literature, software must be made available to editors and reviewers. We strongly encourage code deposition in a community repository (e.g. GitHub). See the Nature Research guidelines for submitting code & software for further information.

Data

Policy information about availability of data

All manuscripts must include a data availability statement. This statement should provide the following information, where applicable:

- Accession codes, unique identifiers, or web links for publicly available datasets
- A list of figures that have associated raw data
- A description of any restrictions on data availability

Raw amplicon sequencing data and shotgun metagenomics sequencing data reported in this study have been deposited in European Genome-phenome Archive with accession codes EGAS00001005651 and EGAS00001005649 respectively.

Field-specific reporting

Please select the one below that is the best fit for your research. If you are not sure, read the appropriate sections before making your selection.

☒ Life sciences ☐ Behavioural & social sciences ☐ Ecological, evolutionary & environmental sciences

For a reference copy of the document with all sections, see nature.com/documents/nr-reporting-summary-flat.pdf

Life sciences study design

All studies must disclose on these points even when the disclosure is negative.

Sample size	No sample size calculation was performed prior to cohort recruitment. This is the first study on multigeneration microbiota transmission and therefore no previous information was available. However, previous studies on mother-infant transmission showed similar sample sizes were sufficient (PMID: 28144631, 30001516, 30001517).
Data exclusions	No data were excluded from the analyses.
Replication	We used a published dataset (Costea et al, 2017) to replicate the main findings of the study.
Randomization	Not applicable: this was a cross-sectional study limited in size, not a randomized study. No intervention was performed on subjects, and therefore no random allocation into groups.
Blinding	This was a data-driven cross-sectional study. As in similar studies, data collection and analysis were not performed blinded to the conditions of the study set-up.

Reporting for specific materials, systems and methods

We require information from authors about some types of materials, experimental systems and methods used in many studies. Here, indicate whether each material, system or method listed is relevant to your study. If you are not sure if a list item applies to your research, read the appropriate section before selecting a response.

Materials & experimental systems

n/a	Involved in the study
☒	☐ Antibodies
☒	☐ Eukaryotic cell lines
☒	☐ Palaeontology and archaeology
☒	☐ Animals and other organisms
☐	☒ Human research participants
☒	☐ Clinical data
☒	☐ Dual use research of concern

Methods

n/a	Involved in the study
☒	☐ ChIP-seq
☐	☒ Flow cytometry
☒	☐ MRI-based neuroimaging

Human research participants

Policy information about studies involving human research participants

Population characteristics	A complete description of the study participants can be found in Table S1.<br>102 generally-healthy female individuals belonging to 24 families ([3:5] generations/family) were enrolled in the study.<br>Age range: [0:98]<br>Vaginal delivery: 99 yes:3 no
Recruitment	The FGFP recruitment channels (social media, newsletters, appearances in popular media) were used to enroll any interested women from families with at least three generations of women in Flanders. All families who volunteered to participated and followed the inclusion criteria were included in the studies, no recruitment bias is expected. Recruitment took place between November 2015 and November 2016.
Ethics oversight	All experimental protocols were approved by the Medical Ethics Committee UZ Brussels-VUB (BUN 143201215505) and the Commissie Medische Ethiek, UZ/KU Leuven (S58125). Study design complied with all relevant ethical regulations, aligning with the Declaration of Helsinki and in accordance with Belgian privacy legislation. Written informed consent was obtained from all adult participants, and from the parents of under-aged participants.

Note that full information on the approval of the study protocol must also be provided in the manuscript.

Flow Cytometry

Plots

Confirm that:

☒ The axis labels state the marker and fluorochrome used (e.g. CD4-FITC).

☒ The axis scales are clearly visible. Include numbers along axes only for bottom left plot of group (a 'group' is an analysis of identical markers).

☒ All plots are contour plots with outliers or pseudocolor plots.

☒ A numerical value for number of cells or percentage (with statistics) is provided.

Methodology

Sample preparation	0.2 g frozen (-80°C) faecal aliquots were dissolved in physiological solution (8.5 g/L NaCl; VWR International, Germany) to a total volume of 100 mL. Subsequently, the slurry was diluted 1000 times. Samples were filtered using a sterile syringe filter (pore size of 5 µm), and 1 mL of the resulting microbial cell suspension was stained with 1 µL SYBR Green I (1:100 dilution in DMSO; shaded 15 min incubation at 37°C; 10,000 concentrate).
Instrument	C6 Accuri flow cytometer (BD Biosciences, New Jersey, USA).
Software	BD Accuri CFlow software v1.0.264.21 (BD Biosciences, New Jersey, USA).
Cell population abundance	Not applicable: no sorting of the fractions was performed.
Gating strategy	Fluorescence events were monitored using the FL1 533/30 nm and FL3 >670nm optical detectors. In addition, forward and sideward-scattered light was collected. The BD Accuri CFlow software was used to gate and separate the microbial fluorescence events on the FL1/FL3 density plot from background. A threshold value of 2000 was applied on the FL1 channel. The gated fluorescence events were evaluated on the forward/sideward density plot, to exclude remaining background events. Instrument and gating settings were kept identical for all samples.

☒ Tick this box to confirm that a figure exemplifying the gating strategy is provided in the Supplementary Information.
